# Emergence and Comparative Genomics Analysis of Extended-Spectrum-β-Lactamase-Producing Escherichia coli Carrying *mcr-1* in Fennec Fox Imported from Sudan to China

**DOI:** 10.1128/mSphere.00732-19

**Published:** 2019-11-20

**Authors:** Chunyan Feng, Peipei Wen, Hao Xu, Xiaohui Chi, Shuang Li, Xiao Yu, Xiangmei Lin, Shaoqiang Wu, Beiwen Zheng

**Affiliations:** a Institute of Animal Quarantine, Chinese Academy of Inspection and Quarantine, Beijing, China; b Collaborative Innovation Center for Diagnosis and Treatment of Infectious Diseases, State Key Laboratory for Diagnosis and Treatment of Infectious Diseases, The First Affiliated Hospital, School of Medicine, Zhejiang University, Hangzhou, China; c Department of Laboratory Medicine, The First Affiliated Hospital of Zhengzhou University, Zhengzhou, China; d Department of Environment and Health, School of Public Health, Shandong University, Jinan, China; Escola Paulista de Medicina/Universidade Federal de São Paulo

**Keywords:** ESBL, *Escherichia coli*, MCR-1, fennec fox, CTX-M-55

## Abstract

The aim of this study was to investigate the occurrence and genomic characteristics of extended-spectrum-β-lactamase-producing Escherichia coli (ESBL-EC) in fennec fox imported from Sudan to China. We screened 88 fecal samples from fennec fox for ESBL-EC, using cefotaxime- and meropenem-supplemented selective medium. Antimicrobial susceptibility testing was performed by the agar dilution method except for colistin and tigecycline; for colistin and tigecycline, testing was conducted by the broth microdilution method. ESBL-EC bacteria were sequenced, and their genomes were characterized. Plasmid conjugation, S1 nuclease pulsed-field gel electrophoresis (PFGE), and Southern blotting were performed for a MCR-1-producing isolate. The genetic environment of *mcr-1* and ESBL genes was also investigated. A total of 29 ESBL-EC bacteria were isolated from 88 fennec fox (32.9%), while no carbapenemase producers were found. The most prevalent genotypes were the *bla*_CTX-M-55_ and *bla*_CTX-M-14_ genes, followed by *bla*_CTX-M-15_ and *bla*_CTX-M-64_. We detected nine sequence types among 29 ESBL-EC. Furthermore, the *mcr-1* gene was detected in isolate EcFF273. Conjugation analysis confirmed that the *mcr-1* gene was transferable. S1 PFGE, Southern blotting, and whole-genome sequencing revealed that *mcr-1* and *bla*_CTX-M-64_ were both located on a 65-kb IncI2 plasmid. This study reports for the first time the occurrence of ESBL-EC in fennec fox. The high prevalence of ESBL producers and the occurrence of MCR-1 producer in fennec fox imported into China from Sudan are unexpected. In addition, it clearly demonstrated that commensal E. coli strains can be reservoirs of *bla*_CTX-M_ and *mcr-1*, potentially contributing to the dissemination and transfer of such genes to pathogenic bacteria among fennec fox. Our results support the implication of fennec fox as a biological vector for ESBL-producing members of the *Enterobacteriaceae* family.

**IMPORTANCE** The extended-spectrum-β-lactamase (ESBL)-producing members of the *Enterobacteriaceae* family are a global concern for both animal and human health. There is some information indicating a high prevalence of ESBL producers in food animals. Moreover, there have been an increasing number of reports on ESBL-producing strains resistant to the last-resort antibiotic colistin with the global dissemination of the plasmid-mediated *mcr-1* gene, which is believed to have originated in animal breeding. However, little is known regarding the burden of ESBL-producing *Enterobacteriaceae* on wild animals. No data were available on the prevalence of antimicrobial resistance (AMR) among wild animals imported into China. This is the first study to investigate the microbiological and genomics surveillance investigation of ESBL colonization among fennec fox (*Vulpes zerda*) imported from Sudan to China, and we uncovered a high prevalence of ESBL-EC. Furthermore, the underlying mechanism of colistin resistance in an isolate that harbored *mcr-1* was also investigated. Results of characterization and analysis of 29 ESBL-producing E. coli may have important implications on our understanding of the transmission dynamics of these bacteria. We emphasize the importance of improved multisectoral surveillance for colistin-resistant E. coli in this region.

## Abstract (precis)

The extended-spectrum-β-lactamase (ESBL)-producing members of the *Enterobacteriaceae* family are a global concern for both animal and human health. There is some information indicating a high prevalence of ESBL producers in food animals. Moreover, there have been an increasing number of reports on ESBL-producing strains resistant to the last-resort antibiotic colistin with the global dissemination of the plasmid-mediated *mcr-1* gene, which is believed to have originated in animal breeding. However, little is known regarding the burden of ESBL-producing *Enterobacteriaceae* on wild animals. No data were available on the prevalence of antimicrobial resistance (AMR) among wild animals imported into China. This is the first study to investigate the microbiological and genomics surveillance investigation of ESBL colonization among fennec fox (*Vulpes zerda*) imported from Sudan to China, and we uncovered a high prevalence of ESBL-EC. Furthermore, the underlying mechanism of colistin resistance in an isolate that harbored *mcr-1* was also investigated. Results of characterization and analysis of 29 ESBL-producing E. coli may have important implications on our understanding of the transmission dynamics of these bacteria. We emphasize the importance of improved multisectoral surveillance for colistin-resistant E. coli in this region.

## OBSERVATION

The increased use of antimicrobial agents in human and veterinary medicine has resulted in an emergence of antimicrobial resistance (AMR) in clinical settings, as well as animal hospitals at large (1). The global spread of extended-spectrum β-lactamases (ESBLs) is of great concern to human and animal health, and CTX-M ESBLs are the most predominant ESBL enzymes worldwide (2, 3). There is increasing evidence that the dissemination of ESBL producers is an issue that is no longer restricted to the clinical service system but represents a growing problem involving animal and environmental integrity (4). In the past few years, ESBL-producing Escherichia coli (ESBL-EC) strains have been reported in healthy animals (5, 6), but the burden of ESBL-producing members of the *Enterobacteriaceae* family on wild animals remain largely undetermined. The dissemination of ESBL genes among wild animals is important to human health because wildlife may act as potential reservoirs and vectors for the spread of AMR (4). No data were available on the prevalence of AMR among wild animals imported into China. Here, we performed a microbiological and genomics surveillance investigation of ESBL colonization among fennec fox (*Vulpes zerda*) imported from Sudan to China.

A total of 168 nonduplicate fresh fecal samples from wild fennec fox imported from Sudan to China were collected from the quarantine yard between August 2017 and May 2018. Permission was obtained from the Entry-Exit Inspection and Quarantine Bureau of China. Individual fresh fecal samples were collected using a sterile swab and subsequently diluted in sterile physiological saline solution as described previously (7). The diluted fecal samples were plated on MacConkey agar supplemented with 1 mg/liter cefotaxime and 1 mg/liter meropenem and incubated for 18 h at 37°C. The resulting strains were then isolated and identified by matrix-assisted laser desorption ionization−time of flight mass spectrometry (MALDI-TOF MS) (Bruker, Bremen, Germany) as described previously (5). Confirmation of ESBL-EC was further performed using a double-disk diffusion method, according to the protocol described by the Clinical and Laboratory Standards Institute (CLSI) (https://clsi.org/). The genes encoding CTX-M type enzymes were screened by PCR followed by Sanger sequencing as reported previously (8).

Susceptibilities to 17 antibiotics (amikacin, amoxicillin-clavulanic acid, aztreonam, cefpirome, cefotaxime, ceftazidime, chloramphenicol, ciprofloxacin, fosfomycin, florfenicol, gentamicin, imipenem, levofloxacin, meropenem, piperacillin-tazobactam, tetracycline, and trimethoprim-sulfamethoxazole) for all ESBL-producing isolates, as well as their transconjugants, were evaluated by the agar dilution method and interpreted according to the CLSI standards, while susceptibilities of isolates to colistin and tigecycline were performed by broth microdilution and interpreted by EUCAST (http://www.eucast.org/) and the U.S. Food and Drug Administration (FDA) breakpoints (9), respectively.

Whole-genome sequencing (WGS) was performed on all 29 ESBL-EC isolates as described previously (5). Briefly, WGS was conducted on an Illumina NovaSeq 6000 platform (Illumina, San Diego, CA, USA) using a Nextera XT library with 2 × 150 paired-end protocol and *de novo* assembled using SPAdes (http://cab.spbu.ru/software/spades/). Multilocus sequence type (MLST) analysis was conducted with WGS data following the MLST database (http://enterobase.warwick.ac.uk/species/ecoli/allele_st_search). Screening for acquired antimicrobial resistance genes and plasmid replicons was performed using the Center for Genomic Epidemiology (CGE) server (http://www.genomicepidemiology.org). Core genomic single nucleotide polymorphism (SNP) was analyzed by the kSNP program (https://omictools.com/ksnp-tool). The maximum likelihood tree of the core SNP matrix output of kSNP was generated by using iTOL (interactive tree of life) (https://itol.embl.de/).

The transferability of plasmids carrying ESBL genes and *mcr-1* gene was determined by filter mating as described previously (10). Southern blotting and S1 nuclease pulsed-field gel electrophoresis (PFGE) analysis were performed to estimate the size of the plasmids harboring ESBL genes and the *mcr-1* gene (11). The sequence of the plasmid carrying *mcr-1* and ESBL genes was generated by plasmidSPAdes (https://omictools.com/plasmidspades-tool). BRIG (BLAST ring image generator) (http://sourceforge.net/projects/brig/) and Easyfig (http://mjsull.github.io/Easyfig/) were used to generate the genetic comparison figures.

A total of 104 Gram-negative isolates resistant to broad-spectrum cephalosporins were cultured by selective medium plates. Of these isolates, 29 isolates from 168 fennec fox fecal samples were identified to be ESBL-EC, giving a carriage rate of 17.3% (29/168). We did not identify carbapenemase-producing E. coli from any sample. Only a few reports have described the antimicrobial use patterns and antimicrobial-resistant bacteria among fur animals (12, 13). To the best of our knowledge, this study reports for the first time the occurrence of ESBL-EC in fennec fox. Previous surveillance data on AMR among *Enterobacteriaceae* from wild animals underscored a wide dissemination of multidrug-resistant (MDR) bacteria, exhibiting an acquired resistance to multiple antibiotic categories (4). On the other hand, previous investigations showed that wildlife act as important role for spreading AMR across the globe (14). The outcome could be explained by the rapid transmission routes and the occurrence of MDR bacteria in potential ecological niches across national boundaries. The risk of zoonotic potential for ESBL-encoding bacteria from importing animals deservedly garners considerable attention.

Among these ESBL-EC strains, total resistance to cefotaxime was observed (100%), followed by very high incidences of resistance to tetracycline (86.2%), ciprofloxacin (72.4%), and trimethoprim-sulfamethoxazole (72.4%) (see Fig. S1 in the supplemental material). All isolates were susceptible to piperacillin-tazobactam, tigecycline, imipenem, and meropenem. Interestingly, only one isolate (EcFF273) was observed to be resistant to colistin. PCR and sequencing further confirmed the presence of the *mcr-1* gene in isolate EcFF273. In this study, the *bla*_CTX-M_ gene was detected in all ESBL-EC strains. The most prevalent genotypes observed in this study were *bla*_CTX-M-55_ and *bla*_CTX-M-14_ gene (*n* = 10 for each), followed by *bla*_CTX-M-15_ (*n* = 8), and *bla*_CTX-M-64_ (*n* = 1). CTX-M-55 became one of the most common ESBL types detected in humans and animals and in the environment in Asian countries (3, 8, 15, 16). Very recently, the occurrence of CTX-M-55-producing E. coli has been increasingly reported in the environment and diverse animal species in Europe (17, 18). Our findings further highlighted that wild animals from North Africa may act as the reservoir of CTX-M-55.

10.1128/mSphere.00732-19.1 FIG S1 Antimicrobial susceptibility of all 29 ESBL-EC strains. Download FIG S1, TIF file, 2.3 MB. Copyright © 2019 Feng et al. 2019 Feng et al. This content is distributed under the terms of the Creative Commons Attribution 4.0 International license.

**FIG 1:**
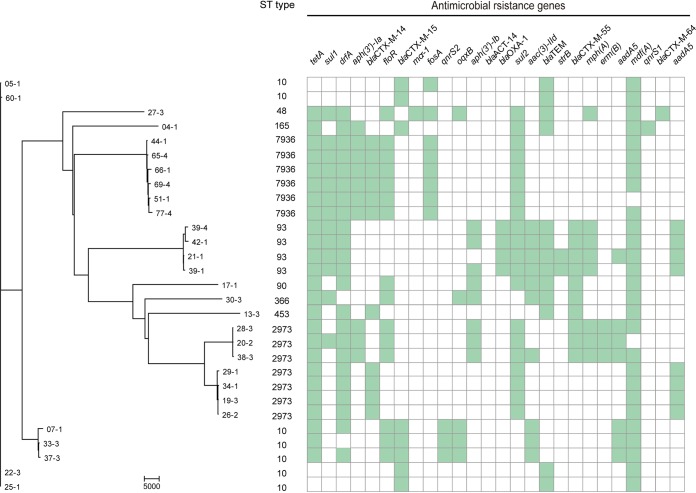
Maximum likelihood core gene phylogeny of 29 ESBL-EC isolates generated by kSNP. Sequence types of ESBL-EC was also indicated. WGS data were uploaded to CGE (http://www.genomicepidemiology.org/) to obtain acquired antimicrobial resistance genes encoded by the isolates. The heatmap is used to display the types of acquired AMR genes. The presence (mint green) and absence (colorless) of AMR genes are indicated.

In this work, we detected nine sequence types (STs) among 29 ESBL-EC, namely, sequence type 10 (ST10) (7/29), ST2973 (7/29), ST93 (4/29), ST90 (1/29), ST48 (1/29), ST165 (1/29), ST366 (1/29), ST453 (1/29), and a new ST (6/29) (ST7936) (Fig. 1). ST10 was the predominant serotype detected among CTX-M-15 producers (7/8) (Fig. S1 and Fig. 1). The presence of ST10 E. coli producing CTX-M-15 has been commonly reported in food animals and companion animals (19, 20). The evidence presented in this study suggests that this lineage has spread to Vulpes zerda. WGS analysis also showed that all isolates harbored a variety of acquired antimicrobial resistance genes (Fig. 1). Of note, seven isolates were observed to carry the *floR* florfenicol resistance gene. A previous investigation found that the *floR* gene is widespread in isolates from food animals and the environment adjacent to the farm (21). Our results underscore the potential wide dissemination of the *floR* gene among ESBL isolates from wild animals.

**FIG 2:**
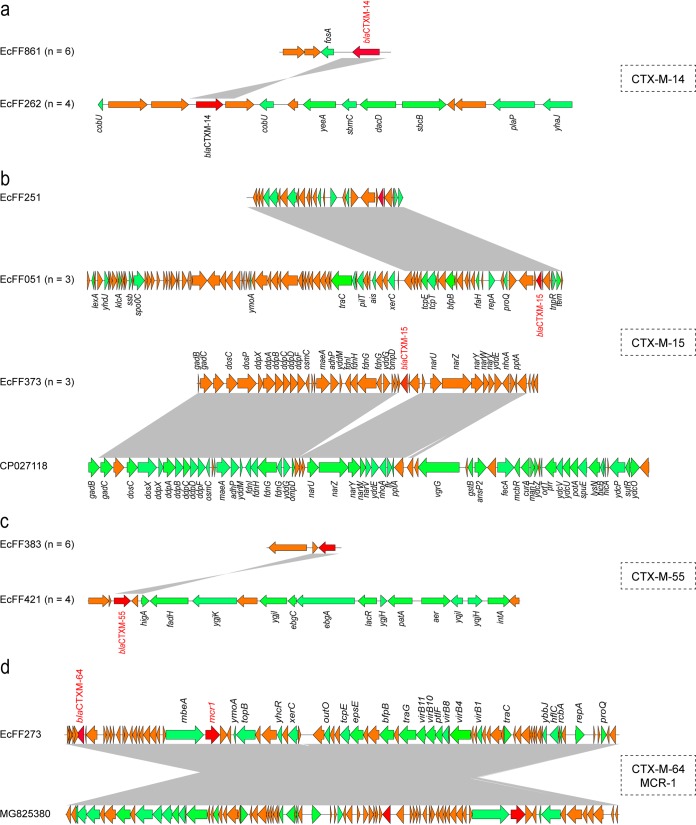
Genetic environment of ESBL genes and *mcr-1* in 29 ESBL-EC isolates. (a) Colinear genome alignment among 10 isolates harboring *bla*_CTX-M-14_. (b) Genetic environment of the *bla*_CTX-M-15_ genes in four ESBL-EC isolates. (c) Genomic map of the *bla*_CTX-M-55_ genes among 10 ESBL-EC isolates. (d) Genetic environment of the *bla*_CTX-M-64_ and *mcr-1* genes in the EcFF273 isolate. The Easyfig program was applied for comparative genomics. Colored arrows indicate open reading frames (ORFs), and the gray-shaded region reflects sequence similarity. The arrows indicate the directions of transcription of the genes. The antimicrobial resistance genes (ARGs) are indicated in red. Isolates with different sizes of the core region of ESBL genes are indicated by vertical lines as well as numbers.

WGS and *in silico* analysis found two types of genetic context surrounding *bla*_CTX-M-14_ (Fig. 2a). In addition, CTX-M-15-producing isolates were genetically divergent, and the genetic structure of *bla*_CTX-M-15_ gene could be categorized into two groups (Fig. 2b). Of note, four ST93 isolates have the same structure of *bla*_CTX-M-55_-encoding fragment (Fig. 2c), which is consistent with the phylogenetic analysis results (Fig. 1). These observations suggest the clonal spread of ST93 among our isolate collection. Interestingly, plasmidSPAdes analysis generated the assembled sequence of the 65-kb IncI2 plasmid pMCR-EcFF273, which is in line with the plasmid size confirmed by the Southern blot and S1 PFGE experiments (Fig. S2). The assembled sequence was subjected to a BLAST search against the nr/nt database. An overall identical identity (99.9%) query showed coverage of 99% to p1108-MCR (GenBank accession no. MG825380) from E. coli of chicken origin in China. Annotation of the plasmid sequence revealed a typical structure surrounding the *mcr-1* gene (*nikA*-*nikB*-*mcr-1-pap2*) in these plasmid contigs (Fig. 2d). Conjugation analysis confirmed that the *mcr-1* gene in EcFF273 was transferable to the recipient cells (data not shown). Although the use of colistin in food animals is widespread, it is rarely connected to wild animals. These data clearly demonstrated that colonized E. coli strains potentially contribute to the dissemination and transfer of *mcr-1* to pathogenic bacteria from fennec fox. Of note, Southern blot and S1-PFGE revealed that 11 ESBL-EC carried ESBL genes on the plasmid and most of the isolates (17/28) carried *bla*_CTX-M_ genes on the chromosome (Fig. S2). Our results are in line with the previous observations of chromosomally integrated ESBL genes occurring in *Enterobacteriaceae* isolates from animals (22, 23).

10.1128/mSphere.00732-19.2 FIG S2 Estimation of plasmid sizes and Southern blot analysis of ESBL-EC isolates. Plasmid profiles of ESBL-EC isolates were determined by using S1 nuclease as the restriction enzyme. XbaI-digested total DNA of Salmonella enterica serotype Braenderup H9812 was used as a size marker (M). Southern blot hybridization was done with a *mcr-1*-specific probe. CTX-M-1, CTX-M-9, and MCR-1 primers were used as probes. Download FIG S2, TIF file, 2.8 MB. Copyright © 2019 Feng et al. 2019 Feng et al. This content is distributed under the terms of the Creative Commons Attribution 4.0 International license.

This study is limited by the relatively small number of fecal samples tested. Further studies involving more fennec fox samples are urgently needed to study the dissemination dynamics of *mcr* and ESBL genes. In addition, it is not yet clear how MCR producers make their way into fennec fox, although this study supports recent findings that MCR-1-producing E. coli appears to be colonizing new hosts. The introduction of *mcr-1*-positive bacteria in fennec fox may be explained by the food chain-based dissemination pathway, and further comprehensive investigation on the transmission of MCR-1 in quarantine yard with “one health” perspective is warranted.

Collectively, this study reports for the first time the occurrence of ESBL-EC in fennec fox. The high prevalence of ESBL producers and the occurrence of MCR-1 producer in fennec fox imported into China from Sudan are unexpected. In addition, it clearly demonstrated that commensal E. coli strains can be reservoirs of *bla*_CTX-M_ and *mcr-1*, potentially contributing to the dissemination of such genes. Our results support the implication of fennec fox as a biological vector for ESBL-EC.

### Data availability.

The Whole Genome Shotgun BioProject for the ESBL-EC isolates has been deposited at DDBJ/EMBL/GenBank under BioProject accession no. PRJNA562084.
