# Supplementary figures and images for: Emergence and Comparative Genomics Analysis of Extended-Spectrum-β-Lactamase-Producing Escherichia coli Carrying mcr-1 in Fennec Fox Imported from Sudan to China
Source: mSphere. 2019 Nov 20;4(6):e00732-19. doi: 10.1128/mSphere.00732-19 (PMC6887861; doi:10.1128/mSphere.00732-19)

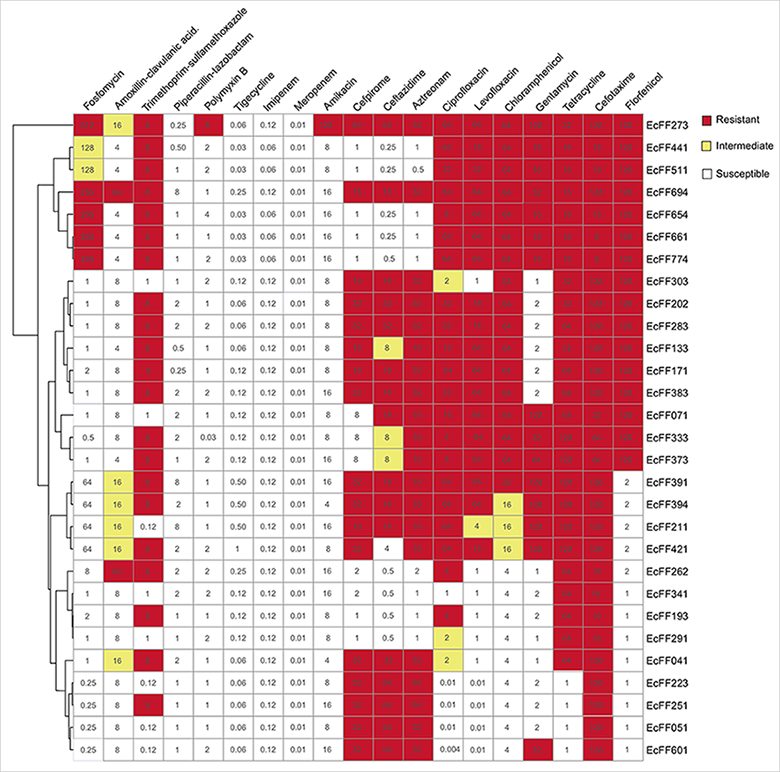

Supplement: FIG S1 [file mSphere.00732-19-sf001.tif]

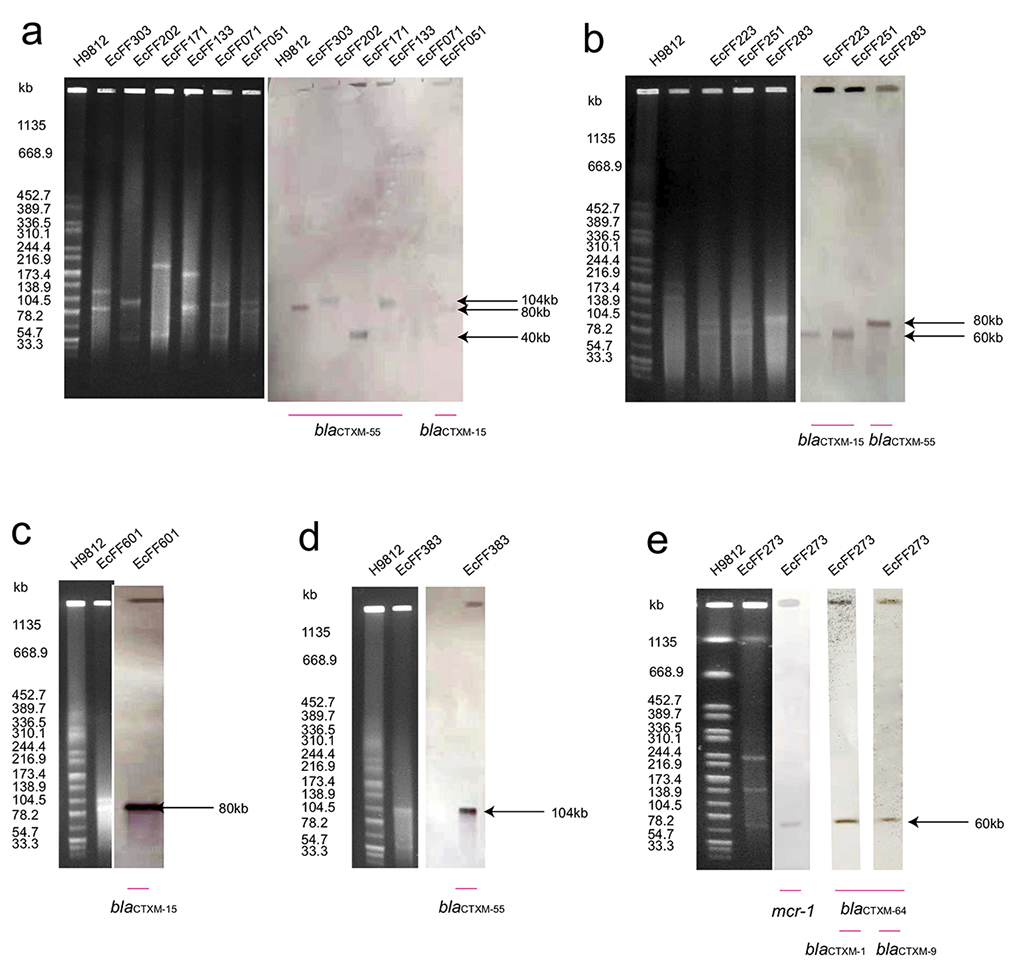

Supplement: FIG S2 [file mSphere.00732-19-sf002.tif]
